# Advances in the role of microRNAs associated with the PI3K/AKT signaling pathway in lung cancer

**DOI:** 10.3389/fonc.2023.1279822

**Published:** 2023-12-19

**Authors:** Yanting Wang, Tao Zhang, Xin He

**Affiliations:** ^1^The First School of Clinical Medicine, Gannan Medical University, Ganzhou, China; ^2^Department of Respiratory and Critical Illness Medicine, Gannan Medical University’s First Affiliated Hospital, Ganzhou, China; ^3^Jiangxi Provincial Branch of China Clinical Medical Research Center for Geriatric Diseases, The First Affiliated Hospital of Gannan Medical University, Ganzhou, China

**Keywords:** microRNA, lung cancer, PI3K/AKT, MDR, therapy

## Abstract

Cancer has long been a topic of great interest in society and a major factor affecting human health. Breast, prostate, lung, and colorectal cancers are the top four tumor types with the greatest incidence rates in 2020, according to the most recent data on global cancer incidence. Among these, lung cancer had the highest fatality rate. Extensive research has shown that microRNAs, through different signaling pathways, play crucial roles in cancer development. It is considered that the PI3K/AKT signaling pathway plays a significant role in the development of lung cancer. MicroRNAs can act as a tumor suppressor or an oncogene by altering the expression of important proteins in this pathway, such as PTEN and AKT. In order to improve the clinical translational benefit of microRNAs in lung cancer research, we have generalized and summarized the way of action of microRNAs linked with the PI3/AKT signaling pathway in this review through literature search and data analysis.

## 1 Introduction

### 1.1 Lung cancer

Lung cancer is the most prevalent carcinoma in the world, which is classified into two distinct kinds based on histology: small cell lung cancer (SCLC) and non-small cell lung cancer (NSCLC). Large cell carcinoma (LULC), squamous cell carcinoma (LUSC), and adenocarcinoma (LUAD) account for approximately 85% of all lung cancer cases. Although SCLC is uncommon, it is distinguished by rapid progression, early metastasis, and a dismal prognosis (1). According to the TCGA database, lung cancer ranks first among male and third among female malignancies by gender, supporting the American Cancer Society’s (ACS) forecast that there will be 236,740 new cases globally in 2022, making up 12.3% of all new cancer cases. LC is the biggest killer of cancer-related deaths globally, causing more than 350 deaths per day, 2.5 times the mortality rate from colorectal cancer (CRC), and more than the sum of the mortality rates from breast, prostate, and pancreatic cancers (2). Clearly, lung cancer has become a serious social burden. Although with the general awakening of people’s health awareness, the early diagnosis rate of lung cancer patients has improved thanks to regular screening of high-risk groups, while the research and development of therapeutic drugs have continued to progress, resulting in an increase in the five-year survival rate of lung cancer patients compared with a decade ago from 17.2% to 21.7% now (3). However, this has not changed the status quo that lung cancer is still the leading cause of cancer deaths, and drug resistance, recurrence and metastasis of tumors are the main reasons that make the overall prognosis of LC patients poorer, with brain metastasis that will lead to rapid death (4). The mechanisms of lung cancer development and progression are complex and are related to extrinsic factors (smoke, dust exposure, etc.) and intrinsic factors (gene mutations, transcript variants), among others (5) (6). The incidence and lethality of lung cancer remain high, so more scientific research is needed to change this cruel status quo. Multiple research efforts have shown that abnormal activity in the PI3K/AKT signaling pathway, which is controlled by a number of endogenous microRNAs, is frequently regarded as a characteristic of the development of cancer.

### 1.2 PI3K/AKT pathway

**Figure 1:**
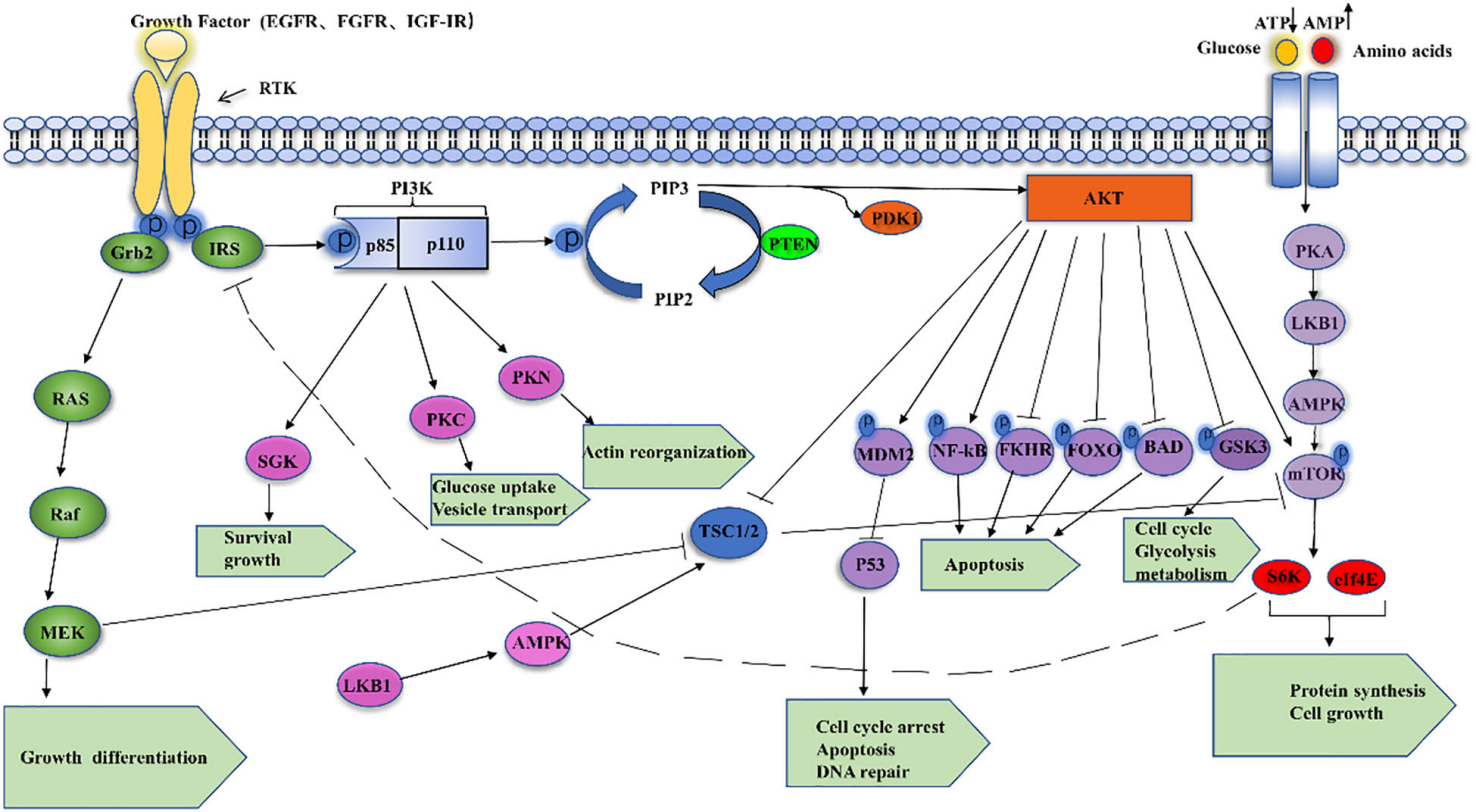
Shows the PI3K/AKT signaling cascade schematically.

The phosphatidylinositol-3 kinase (PI3K)/AKT signaling pathway is activated in response to the membrane receptor tyrosine kinase (RTK) and transmits signals from the cell membrane to the nucleus. RTKs include various types of growth factors, such as the epidermal growth factor receptor (EGFR), insulin-like growth factor I receptor (IGF-IR), and fibroblast growth factor receptor (FGFR) (7) PI3K is a family of lipid kinases with both serine/threonine (Ser/Thr) kinase activity and phosphatidylinositol kinase activity, which possesses the ability to phosphorylate the 3’-OH moiety of the inositol ring in inositol phospholipids (8). The currently known PI3Ks are divided into three classes: class I (α, β, γ, δ), class II (C2α, C2β, C2γ), and class III PI3K VPS34 (also known as PIK3C3), of which class I PI3Ks are heterodimers consisting of the p110 catalytic subunit and the p85 regulatory subunit with the SH2 structural domain. p110 catalytic subunit contains four isoforms (The p110 catalytic subunit contains four isoforms (p110α, p110 β, p110γ, and p110δ, encoded by PIK3CA, PIK3CB, PIK3CG, and PIK3CD, respectively), of which p110γ and p110δ are restricted to leukocytes, whereas the remaining types of catalytic subunits are widely distributed in various cell types (9). Functionally, the p110 catalytic subunit converts PIP2 (phosphatidylinositol 2 phosphate) to PIP3 (phosphatidylinositol 3 phosphate) by binding to the p85 regulatory subunit, which in turn recruits oncogenic signaling proteins with its binding site, including protein kinase B and phosphatidylinositol-dependent protein kinase 1 (PDK1) (10). The cancer suppressor gene PTEN can halt the conversion of PIP2 to PIP3 by acting as an antagonist of PI3K (11). Protein kinase B (Akt), also known as PKB or Rac (12), can encode serine/threonine enzymes, which is an important kinase involved in a variety of physiological activities such as cell proliferation and apoptosis, and phosphorylated Akt can integrate a variety of cellular regulators to promote cancer progression. In addition, phosphorylated Akt can reverse the inhibitory effect of the oncogene TSC1/2 on its downstream effector mTOR, which integrates many of its downstream proteins (S6K, 4EBP1, etc.) to promote cancer progression (13). In the last few decades, the PI3K/Akt signaling pathway has been assumed as being dysregulated in a wide range of human malignancies, with kinase mutations and/or decreased PTEN expression leading to tumor transformation.Cancer cells’ increasing reliance on PI3K/Akt signaling makes it an attractive therapeutic target. Thus, a better knowledge of the mechanisms that regulate aberrant PI3K/Akt signaling in cancer can provide important insights for the development of new therapeutic methods (**Figure 1**).

### 1.3 MicroRNA

MicroRNAs (miRNAs), a subclass of endogenous small molecules non-coding RNAs (ncRNAs), control the activity of protein-coding genes post-transcriptionally (14). The majority of microRNA genes are transcribed by RNA polymerase II to produce stem loops containing primary microRNAs (pri-miRNAs), which can range in size from hundreds to thousands of bases, a small number of miRNAs, contained within Alu repeat elements, can be transcribed by RNA polymerase III (15). Following capping and polyadenylation in the nucleus, primary miRNAs are then cut into short fragments of about 60–70 nucleotides in length by the RNAse III enzyme Drosha, these fragments are then altered by the RNAse II endonuclease Dicer to create mature microRNA duplexes (16). As many as 2300 distinct microRNAs have been identified in human cells, with time- and tissue-dependent expression patterns, and mature microRNAs account for about 1% of the genomes of different species, binding the 3’UTR sequence motifs of mRNAs through partial complementarity and inducing mRNA degradation and translational repression (17) (18), affecting multiple biological functions of cells. Recent studies have found that microRNAs acting on some PI3K/AKT pathway proteins play a role in cancer formation (19).

We observed that several microRNAs regulate the activity of a number of critical proteins in the PI3K/AKT signaling pathway, which influences the overall lung cancer growth process. The paper analyzes and describes the mechanisms of action of these microRNAs in an effort to boost the clinical translational benefits of microRNAs in LC research (**Tables 1**–**3**).

**Table 1:** Up-regulated expression in lung cancer and their target molecules and functions.

MicroRNAs	Expression	Target	Function	Refs
miR-425-5p	↑	PTEN	+	(20)
miR-4507	↑	TP53	+	(21)
MiR-629	↑	FOXO1	+	(22)
miR-141	↑	PHLPP1/PHLPP2	+	(23)
MiR‐4286	↑	PTEN	+	(24)
miR-200	↑	IRS-1	+	(25)
miR-210	↑	RUNX3/UPF1/PTEN	+	(26)
MiR-210-3p	↑	CELF2	+	(27)
miR-212-5p	↑	Id3	+	(28)
miR-21	↑	PTEN、PP2A	+	(29)
miR-142-5p	↑	PTEN、CD4+ T	+	(30)
miR-125b	↑	p-Akt	+	(31)
MiR-93	↑	LKB1/PTEN/CDKN1A	+	(31)
miR-210	↑	UPF1,PTEN	+	(32)
miR-92a	↑	PTEN	+	(33)
miR-224	↑	PTEN	+	(34)
miR-20a	↑	PTEN	+	(35)
miR-199a-5p	↑	p62	+	(36)
miR-522-3p	↑	p-EGFR、 p-AKT	+	(37)
miR-135a	↑	RAC1	+	(38)
miR-23a	↑	PTEN	+	(39)
miR-410	↑	PTEN	+	(40)
miR-424-3p	↑	PTEN	+	(41)
miR-25-3p	↑	PTEN	+	(42)
miR-514b-5p	↑	PI3K,P38	+	(43)

↑, increased; ↓, decreased; +, Activation PI3K/AKT pathway; –, Inactivated PI3K/AKT pathway.

**Table 2:** Dual-action microRNAs in lung cancer and their target molecules and functions.

MicroRNAs	Expression	Target	Function	Refs
miR-17- 5p	↑	PTEN	+	(44)
miR-17- 5p	↓	RRM2	–	(45)
miR-374a	↑	PTEN	+	(46)
miR-374a	↓	CCND1	–	(46)

↑, increased; ↓, decreased; +, Activation PI3K/AKT pathway; –, Inactivated PI3K/AKT pathway.

**Table 3:** Down-regulated microRNAs in lung cancer and their target molecules and functions.

MicroRNAs	Expression	Target	Function	Refs
miR-126	↓	PIK3R2/VEGF/MRP1	–	(47)
miR-1	↓	PIK3CA	–	(48)
MiR-34a	↓	PTEN、YY1、MET	–	(49)
miR-320a-3p	↓	PI3K、p-AKT、ELF3	–	(50)
miR-409	↓	p-AKT、SPIN1	–	(51)
miR-4299	↓	PTEN	–	(52)
MiR-503	↓	PI3K、p85、	–	(53)
miR-496	↓	PI3K、p-AKT、BDNF	–	(54)
MiR-107	↓	p-AKT、BDNF、Bcl-w	–	(55)
miR-217	↓	PI3K	–	(56)
miR-520a-3p	↓	PI3K	–	(57)
miR-448	↓	PI3K、p-AKT、EPHA7	–	(58)
miR-101-3p	↓	PI3K、p-AKT、MALAT-1	–	(59)
miR-381	↓	PI3K、p-AKT、LMO3	–	(60)
miR-519d	↓	PI3K、HER3	–	(61)
miR-519d-3p	↓	p-AKT、VEGF	–	(62)
miR-138-1*	↓	p-AKT、PDK1	–	(63)
miR-379-5p	↓	p-AKT、ARRB1	–	(64)
miR-139-5p	↓	p-AKT、Caspase-3、HOXB2	–	(65)
miR-29a	↓	PTEN、YY1、N-cadherin	–	(33)
miR-4458	↓	p-AKT、HMGA1	–	(66)
miR-122	↓	p-AKT、IGF1R	–	(67)
miR-485	↓	PI3K、p-AKT、FLOT2	–	(68)
miR-20a	↓	PI3K、RRM2	–	(69)
miR-449a	↓	PTEN、NNMT	–	(70)
miR-29c	↓	AKT2	–	(71)
MiR-107	↓	p-AKT、Bcl-w	–	(72)
miR-30a-5p	↓	p-AKT、EGFR、IGF-1R	–	(73)
miR-200c	↓	p-AKT、ZEB1	–	(74)
miR-223	↓	IGF1R、p-AKT	–	(75)
let-7c	↓	RAS	–	(76)
miRNA-126	↓	p-AKT	–	(77)
miR-7	↓	EGFR、AKT	–	(78)
miR-30-5p	↓	PI3K-SIAH2	–	(79)
miR-486-5p	↓	p85	–	(80)
miR-30b-5p	↓	p-AKT、PTEN	–	(81)
miR-30c	↓	PI3K、p-AKT	–	(82)
miR-192-5p	↓	AKT、PI3K	–	(83)
miR-539	↓	ERK	–	(84)
let-7c-3p	↓	PIK3CA	–	(85)
miR-485-5p	↓	PI3K、p-AKT	–	(86)

↑, increased; ↓, decreased; +, Activation PI3K/AKT pathway; –, Inactivated PI3K/AKT pathway.

## 2 MicroRNA affects lung cancer development through the PI3K/AKT pathway

### 2.1 Regulates the growth of lung cancer cells

Loss of control over cell proliferation is the primary hallmark of tumor start, and uncontrolled cell proliferation is regulated by a wide range of factors. It is impossible to ignore the critical regulatory roles that microRNAs play in the progression of many malignancies via the PI3K/AKT signaling pathway. These microRNAs may act as tumor suppressors or cancer-causing genes, influencing the occurrence of human malignancies.

For instance, miR-425-5p, which is overexpressed in NSCLC and enhances the PI3K/AKT signaling pathway by reducing PTEN, promotes the formation of lung cancer (20). Similar to this, miR-4507 overexpression in NSCLC tissues and cells decreased the expression of its potential target TP53 and turned on the PI3K/AKT signaling pathway, which aided in the proliferation of tumor cells (21). It was recently observed that clients who had elevated miR-629 levels usually had poor prognoses, and that high miR-629 levels in NSCLC accelerated NSCLC growth by blocking the transcription factor FOXO1 (22). MiR-141 dramatically boosted the level of phosphorylated AKT in NSCLC cells while deregulating the inhibitory effects of PHLPP1 and PHLPP2, which block the PI3K/AKT signaling pathway and slow NSCLC cell proliferation (23). By controlling the PI3K/Akt pathway and targeting PTEN, miR-4286 accelerates the development of lung cancer cells (24). MiR-200 is elevated in adenocarcinoma and accelerates lung adenocarcinoma tumor cell growth by activating AKT in cells via IRS-1 (25). It was recently shown that miR-210 upregulation promotes the development of NSCLC by controlling the PI3K/AKT signaling pathway and dwelling on the transcription factor RUNX3 (26) ADDIN. MiR-210-3p increased cell proliferation in LUSC by upregulating PI3K/AKT activity and suppressing CELF2 expression (27). Interestingly, miR-374a was shown to play a dual role in NSCLC, miR-374a exerted a protective effect by inactivating PI3K/AKT and RAS-mediated cell cycle signaling, as well as epithelial-mesenchymal transition (EMT) through direct targeting of CCND1 in the early stage of NSCLC. MiR-374a reduced cell growth substantially, which also improved NSCLC cell sensitivity to cisplatin (DDP) and enhanced the survival period of tumor-bearing mice. However, in advanced NSCLC, miR-374a had the opposite impact via targeting PTEN, and the findings suggest that the same miRNA might appear to play distinct functions in various diseases or stages of the same disease (28)ADDIN. A tumor suppressor gene Id3 was found to be associated to the progression of certain malignancies, and in lung cancer studies, researchers discovered that miR-212-5p promotes NSCLC growth and xenograft tumor creation *in vivo* by decreasing Id3 level and activating the PI3K/Akt pathway (46).

In contrast, the expression of some other miRNAs with tumor suppressor effects was reduced in NSCLC. For example, miR-126 expression was markedly downregulated in NSCLC,miR-126 overexpression, which targets PIK3R2 and deactivates the PI3K-Akt signaling pathway, inhibits LUAD cell proliferation as well as tumor growth rate and size in xenograft tumor models (47) (87)ADDIN.By obstructing the PI3K/Akt pathway, miR-1 reduces the tumorigenicity of NSCLC cells in a xenograft tumor model (48). MiR34a was reduced in NSCLC cells, and increased miR34a induced upregulation of PTEN and YY1, inhibition of CDK6 and inactivation of the PI3K/AKT pathway, thereby impeding tumor cell growth (49). MiR-320a-3p’s expression was markedly downregulated in NSCLC tissues and cells, and cell function studies supported the hypothesis that it functioned as a tumor suppressor gene to prevent cell development by downregulating ELF3 and subsequently deactivating the PI3K/Akt pathway (50) (88). MiR-409, a tumor suppressor gene, is less expressed in NSCLC tissues and cells and inhibits the advancement of NSCLC by disrupting the PI3K/AKT pathway by minimizing SPIN1 (51). Similarly, miR-4299 expression was reduced in NSCLC tissues and cells, especially during disease progression, and overexpression of miR-4299 could inactivate the AKT/PI3K pathway to exert tumor suppressor effects (52). MiR-503 inhibits NSCLC progression by altering the expression of PI3K, p85, IKK-b, and PDK1 and blocking the PI3K/AKT pathway (53) (89). MiR-496 has diminished expression in a wide range of NSCLC cells, and researchers have experimentally demonstrated that overexpression of miR-496 could inactivate the BDNF (a brain-derived neurotrophic factor)-mediated PI3K/Akt signaling pathway to inhibit tumor growth (54). In a separate study, miR-107 expression was downregulated in NSCLC, and raising it prevented the BDNF-mediated PI3K/AKT pathway from being activated and limiting cell growth (55). The level of miR-217 expression was dramatically decreased in NSCLC tissues and cells, and it was discovered that upregulation of miR-217 may prevent NSCLC growth and progression by limiting the production of proteins involved in the PI3K pathway (56). MiR-520a-3p is a gene with tumor-suppressive effects in numerous distinct solid tumors, and there is evidence that inhibit NSCLC growth by inactivating the PI3K/AKT/mTOR signaling pathway (57). Similarly, miR-448 expression was downregulated in NSCLC and lower level was linked to a worse patient prognosis; however, miR-448 upregulation slowed the progression of NSCLC due to inhibiting EPHA7 and blocking the PI3K/AKT signaling pathway (58). In NSCLC cells, the overall level of miR-101-3p was markedly decreased, cell growth was decreased by miR101-3p because it prevented the PI3K/AKT signaling pathway from being activated by MALAT-1 (59). MiR-381 was significantly down-regulated in LUAD tissues, and overexpression of miR-381 would inhibit the PI3K/Akt pathway activation and EMT process, thus significantly limiting LUAD cell growth and tumor formation *in vivo (* 60). MiRNAs are found not just in diverse tissues and cells, but also in a number of bodily fluids. MiR-519d, whose levels was downregulated in the blood plasma of NSCLC patients, was discovered to limit the progression of the disease via inhibiting HER3 and deactivating the PI3K/Akt pathway (61). Neovascularization is well established to offer vital nutritional security for the survival and multiplication of tumor cells. It was discovered that overexpression of miR-519d-3p will control angiogenesis in NSCLC by inhibiting the P38 and PI3K/AKT pathways by targeting VEGF (62).

It is widely accepted that environmental toxins like smoke and dust can cause LC. AFB1 has been shown to be able to cause malignant transformation in immortalized human bronchial epithelial cells that are stably expressing CYP2A13; however, overexpression of miR-138-1* has been shown to be able to overcome this event. Mechanistically, miR-138-1* primarily performs the above role by inhibiting the PI3K/PDK/Akt pathway in the generation of PDK1 and its associated proteins to exert the above (63). Meanwhile, miR-21 was discovered to be significantly produced by DEP-stimulated human bronchial epithelial (HBE) cells and increased PI3K/AKT pathway stimulation, which might represent an essential lung carcinogenesis mechanism (90).

It is not difficult to find that these miRNAs, as oncogenes or tumour suppressor genes, affect the activity of the PI3K/AKT pathway by inhibiting or promoting the expression of their downstream target genes, thus regulating the proliferation and differentiation of tumour cells, which is related to the overall prognosis of the whole disease. Through in-depth study of these relevant miRNAs and their mechanisms of action, the future can be achieved through a variety of emerging technologies based on Through in-depth study of these miRNAs and their mechanism of action, it is possible in the future to regulate the expression of relevant miRNAs and change their effects on lung cancer cells through various emerging technologies based on molecular biology, such as genetic programming, so as to inhibit or change the developmental process of lung cancer.

### 2.2 Regulation of apoptosis in lung cancer cells

Apoptosis is a key pathway for regulating cell survival. Cancer development is often significantly characterized by inhibition of apoptosis (91). It was already demonstrated that the PI3K/AKT signaling pathway plays a crucial regulatory function in apoptosis (92), and this regulation is affected by the aberrant expression of many microRNAs. Some of these microRNAs reduce carcinogenesis or promote apoptosis by targeting the mRNAs of proto-oncogenes and shutting them down, while others enhance the anti-apoptotic ability of tumor cells by down-regulating the expression of tumor suppressor genes. For instance, the cancer suppressor gene PTEN is controlled by a variety of microRNAs and affects the PI3K/AKT signaling pathway, which in turn affects the apoptotic process of tumor cells, it was proved that the inactivation of PTEN is a single of the important factors in the development of lung cancer (93). MiR-142-5p was up-regulated in NSCLC tissues and cells. miR-142-5p high expression down-regulated PTEN and induced the activation of PI3K and p-Akt, which inhibited apoptosis and promoted the development of NSCLC (30). MiR-21 expression is upregulated in multiple types of malignant tumors, and in lung cancer, miR-21 inhibits apoptosis in NSCLC cells through activation of the PI3K/Akt pathway (29). MiR-125b was highly expressed in NSCLC tissues, and down-regulation of miR-125b induced apoptosis in NSCLC cells by inactivating PI3K/Akt pathway-related proteins (31). In contrast, miR-379-5p expression was found to be significantly down-regulated in lung cancer, and elevated levels of miR-379-5p would result in reduced levels of phosphorylated PI3K and p-AKT, in addition to inhibit the growth of cells and promote cell death via direct targeting of ARRB1, a scaffolding protein that mediates the desensitization and internalization of G protein-coupled receptors (GPCR) (64). MiR-139-5p is found in low levels in NSCLC tissues and cells, and its overexpression induces apoptosis by inhibiting the PI3K/AKT/caspase-3 signaling pathway (65).

Apoptosis is known to be an important factor in the regulation of cell growth and proliferation rates as well as cancer development. In addition, apoptosis of tumour cells also affects their sensitivity to tumour therapeutic drugs. MiRNAs are involved in the regulation of apoptosis process by affecting the expression of apoptosis-related proteins in the PI3K/AKT pathway. An in-depth study of the mechanism of these miRNAs in apoptosis of tumour cells not only can delay or inhibit the development of tumours, but also improve the therapeutic effect of tumour therapeutic drugs.

### 2.3 Blocking or promoting metastasis of lung cancer

Tumor metastasis involves a complex multi-stage process (including tumor cell detachment from the primary tumor, internal invasion, circulatory spread, extravascular migration, adaptation to the external microenvironment, and organ-specific colony formation) and is closely related to the death of cancer patients (94). MicroRNAs, as key regulatory molecules, regulate their expression and play a significant influence in tumor metastasis through partial complementary binding to mRNAs. As an instance, miR-17-5p expression was found to be upregulated in NSCLC cells with bone metastasis, which promotes osteoclastogenesis by targeting the PTEN-activated PI3K/Akt pathway in lung cancer, and treatment with LY 294002, an inhibitor of the PI3K/Akt pathway, inhibited miR-17-5p-mediated osteoclastogenesis (44). In another study, researchers found that high expression of miR-93 not only promoted NSCLC cell growth, but also induced significant liver metastasis of lung cancer in a mouse xenograft tumor model (95). Tumor-mesenchymal interactions are crucial for cancer development and metastasis, and tumor-associated cells (CAFs) are one of the major components of the tumor mesenchyme, whereas exocytosis (Exo) is a type of microvesicles secreted by living cells that mediate intercellular communication, and in recent years, it has been found that CAFs promote tumor metastasis by carrying Exo to neighboring cells (96). In studies related to NSCLC, miR-210 was demonstrated to be highly expressed in CAFs-exo and promoted the EMT process in NSCLC cells by down-regulating UPF1 and PTEN, as well as activating the PI3K/AKT pathway thus promoting the EMT process in NSCLC cells (EMT refers to the loss of its epithelial properties by epithelial cells and the adoption of a mesenchymal-like phenotype, and a number of studies have been conducted to confirm that EMT is a tumor metastatic key link) (32). Similarly, miR-92a was shown to promote NSCLC metastasis by down-regulating PTEN and activating the PI3K/AKT signaling pathway, thereby inducing the EMT process in NSCLC cells (33). On the contrary, miRNA-126-loaded exosome 231-Exo not only recognizes A549 cells in the blood to inhibit their growth, but also induces lung homing effect of tumors in a mouse lung cancer metastasis model. mechanistic studies confirmed that miRNA-126-loaded exosome 231-Exo inactivates the PI3K/AKT signaling pathway through down-regulation of PTEN to produce the above effects (97). Similarly, it was found that overexpression of miR-29a could block the IL-13-induced invasive process in A549 cells by inactivating the PI3K/AKT/AKT/YY1 axis (98).MiR-4458 expression was observed to be decreased in NSCLC cells, and miR-4458 increased levels inactivated the PI3K/AKT signaling pathway, limiting NSCLC migration and EMT progression (66). MiR-122 inhibits metastasis and EMT in NSCLC cells via inhibiting the PI3K/AKT signaling pathway by lowering IGF1R expression (67). MiR-485, a tumor suppressor gene, is lowly expressed in LUAD. miR-485 has been found to inhibit LUAD metastasis and EMT by down-regulating FLOT2 to inactivate the PI3K/Akt/mTOR signaling pathway (68). Angiogenesis is a major element in the recurrence and spread of cancer, and ribonucleotide reductase regulatory subunit M2 (RRM2) has been provided to play a key control effect in restricting the growth of tumor capillaries. According to a lung cancer study, miR-20a-5p suppresses NSCLC angiogenesis and metastasis via blocking RRM2 and inactivating the PI3K/Akt pathway (69).

Metastasis is the most important feature of malignant tumors and a major factor in the poor prognosis of patients with advanced lung cancer. EMT is an important process that determines the fate of tumor cells and influences their malignant metastasis, and miRNAs regulate the EMT process by affecting the expression of related proteins in the PI3K/AKT signaling pathway, suggesting that blocking lung cancer cell metastasis by regulating the expression of these miRNAs may be a new therapeutic idea.

### 2.4 Regulation of tumor metabolism in lung cancer

For a long time in the past, it seemed to be a consensus that tumor cells needed more nutrients to promote their rapid proliferation. However, with the increasing research on tumor metabolism, it has been found that unlike normal tissue cells that produce energy by mitochondrial oxidative phosphorylation, tumor cells rely on aerobic glycolysis, an inefficient energy pathway, to provide energy for their own growth and proliferation despite the presence of oxygen, which has also been termed as the “Wahlberg effect “ (99). Some studies have confirmed that this tumor metabolic mode promotes the growth of tumor cells while it is more conducive to their survival in the unfavorable conditions of the tumor microenvironment (100). The tumor metabolic mode is related to the tumor’s own growth characteristics, and further understanding of the mechanistic link between tumor cell metabolism and growth control will help to seek better tumor therapies. Notably, microRNAs, as the most widely studied star molecules, are involved in regulating the aerobic glycolysis process in tumors through the PI3K/AKT pathway, which facilitates the process of tumor drug elimination while altering tumor metabolism (101).

For instance, it was discovered that overexpression of the miR-449a gene would down-regulate nicotinamide n-methyltransferase (NNMT), a tumor-metabolizing enzyme, and cause an increase in the expression of PTEN, which would inhibit tumor growth, in EGRR-AKT resistant NSCLC cells. Additionally, the researchers showed that the natural anti-tumor medication Yuanhuadine (YD) greatly increased miR-449a levels, which prevented NNMT production (70). According to this study, developing anticancer medications may benefit from a deeper understanding of how microRNAs regulate tumor metabolism by activating the PI3K/AKT signaling pathway.

### 2.5 Regulating the tumor microenvironment in lung cancer

Alterations in the tumor microenvironment (TME) can affect several pathophysiological processes such as tumor growth and metastasis, suggesting that the occurrence of solid tumors is not only related to genetic mutations but also to alterations in the environment in which cells live (102). According to one study, the downstream molecule of the PI3K/Akt signaling pathway, mechano/mammalian target of rapamycin (mTOR), can be involved in the regulation of multiple physiological functions of tumor cells by integrating various cellular signals in the TME (103). In a related study of lung cancer, researchers found that TME nutrient deficiency promoted the transfection efficiency of miR-224 mimics in NSCLC cells, in addition to altering the expression of Bcl-2, PTEN, apoptotic protein Bax, and autophagy-associated protein LC3 and affecting changes in the corresponding cellular functional phenotypes, a finding that suggests that alterations in TME have a critical impact on tumors (34).

### 2.6 Modulating multidrug resistance in lung cancer

**Figure 2:**
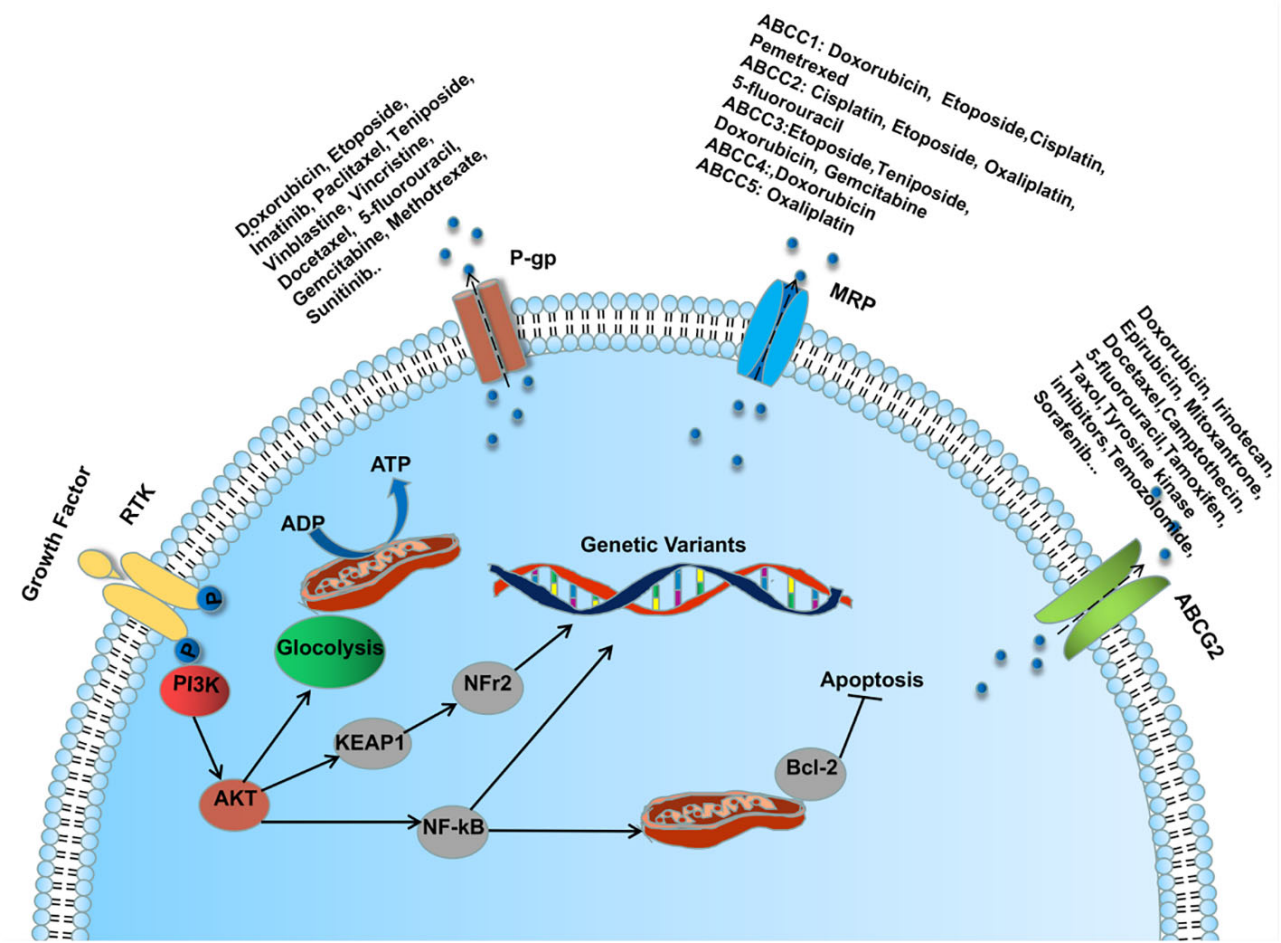
MicroRNA regulates ABC transporter through PI3K/AKT pathway involved in the mechanism of MDR formation during lung cancer drug therapy.

Lung cancer patients are treated with surgery, platinum-based chemotherapy combined with radiotherapy, molecular biology-based immunotherapy and molecularly targeted therapy, which to some extents have brought benefits to patients with primary lung cancer. Unfortunately, with the emergence of multidrug resistance (MDR), patients do not have a favorable outcome of advanced drug therapy and have a poor overall prognosis. MDR is a phenomenon in which a patient develops resistance to the given drug and other structurally similar drugs during drug therapy (104) ADDIN. The emergence of MDR involves many mechanisms, one of which is classically dependent on the ATP-binding cassette (ABC) transporter (P- gp, MRP1 and BCRP) mediated drug efflux (105). (**Figure 2**) Another important mechanism is to disrupt apoptosis or alter the cell cycle by regulating the aberrant expression of relevant cytokines, which promotes tumor cell proliferation and makes tumor cells resistant to drug-induced cell death and cell cycle block (106). In recent years, studies on drug resistance in lung cancer have confirmed that the aberrant expression of endogenous miRNAs promotes or inhibits the production of MDR during LC drug therapy by regulating the ABC transporter of the PI3K/AKT pathway, the expression of apoptosis-associated proteins, nuclear factor κb (NF-κB), glycogen synthase kinase 3β (GSK-3β), and mTOR, among others (107). Therefore, further investigation of the regulatory mechanisms of miRNAs in MDR production may bring new hope to those drug-resistant lung cancer patients.

In lung cancer chemoresistance studies, miR-139-5p enhanced the chemosensitivity of NSCLC by inducing apoptosis and reversing the phenomenon of cisplatin (DDP)-induced cellular resistance through inhibiting the PI3K/AKT/caspase-3 pathway (65). In the same way, it turned out that miR-29c knockdown increased cellular drug resistance whereas miR-29c upregulation improved NSCLC cells’ sensitivity to DDP, this difference was linked to miR-29c’s modulation of the PI3K/Akt pathway (71). Exosome-derived miR-20a induces DDP resistance in lung cancer cells by downregulating PTEN and promoting the PI3K/AKT pathway (35). Previous studies have shown that the expression of miR-25-3p is up-regulated in cisplatin-resistant NSCLC cells, and enhance cisplatin resistance by regulating PTEN/PI3K/AKT signaling pathway, and inhibition of miR-25-3p may become a new strategy to overcome cisplatin resistance in NSCLC (42).

In addition to platinum-based chemotherapeutic agents, the production of MDR also affects the efficacy of other adjuvant chemotherapeutic agents. For example, it has been discovered that after receiving paclitaxel (PTX), lung cancer cells’ reactivity to PTX declines as the levels of level of miR-199a-5p rises. Mechanistically, miR-199a-5p inhibits the expression of autophagy-related proteins by activating the PI3K/Akt/mTOR pathway, which promotes the development of MDR in lung cancer cells (36). Similarly, miR-107 enhances chemosensitivity of NSCLC to PTX by down-regulating the anti-apoptotic factor Bcl-w (72). MiR-126 is under-expressed in NSCLC cells, and overexpression of miR-126 inactivates PI3K/Akt signaling by targeting vascular endothelial growth factor A (VEGFA) and multidrug resistance-related protein 1 (MRP1) pathway and induced a significant reduction in the maximal inhibitory concentrations of adriamycin (ADM) and vincristine (108). MiR-17-5p is lowly expressed in gemcitabine-resistant cell lines (A549/G+ cells), and increased expression of miR-17-5p would reverse gemcitabine resistance in this cell (45).

Epidermal growth factor receptor tyrosine kinase inhibitors (EGFR-TKIs), which are frequently employed in molecular targeted therapy for lung cancer, have demonstrated good performance (109). Unfortunately, some NSCLC patients have successively developed secondary EGFR T790M mutations, receptor tyrosine kinase c-MET (MET), and hepatocyte growth factor (HGF) overexpression after receiving long-term treatment, leading to acquired resistance thus limiting the efficacy of EGFR-TKIs (110) (111) (112). To add to the grim reality, it has been reported that approximately 61% of patients with acquired resistance to TKIs were detected with HGF overexpression (113). HGF is a ligand for the MET oncoprotein, which induces drug resistance in EGFR-mutated NSCLC cells by phosphorylating MET (112). Increasing evidence suggests that miRNAs are key regulatory molecules that modulate the sensitivity of tumor cells to EGFR-TKIs. For example, miR-34a can reverse HGF-mediated gefitinib resistance by partially targeting MET (114). In addition, it has been demonstrated that the horizontal transmission of drug-resistant phenotypes between cells can be achieved through a certain pathway, which results in the emergence of drug resistance or reduced drug sensitivity in cells that are originally sensitive to antitumor drugs. For example, T790M-mutated EGFR-TKIs-resistant cells release exosomes encapsulating miR-522-3p to activate the PI3K/AKT signaling pathway thereby inducing resistance to gefitinib in sensitive cells (37). MiR-30a-5p in combination with other EGFR-TKIs will increase the sensitivity of NSCLC cells to gefitinib (73) (115). Similar to this, miR-200c improved NSCLC cells’ susceptibility to gefitinib by obstructing the PI3K/Akt signaling pathway (74).MiR-223 expression was significantly reduced in the TKI-resistant cell lines PC9/ER and PC9/CD133+ cells, and overexpression of miR-223 would attenuate the cellular expression of IGF1R mRNA and p-Akt levels, thus restoring the sensitivity of resistant cells to erlotinib (75). MiR-135a induces resistance to gefitinib in NSCLC cells through RAC1 (a member of the Rho family of GTPases) and PI3K/Akt signaling pathways, whereas knockdown of miR-135a reverses the above resistance phenomenon (38). Interestingly, a number of research in recent years have discovered that the estrogen receptor ERB works as a mitogen in NSCLC cells, and that the bidirectional signaling loop between the estrogen and EGFR pathways increases tumor-associated angiogenesis while accelerating NSCLC growth, furthermore, let-7c is significantly up-regulated in anti-estrogenic (fulvestrant)-treated cells, and let-7c increases gefitinib sensitivity via RAS inhibition, PI3K/AKT inactivation, and the mitogen-activated extracellular signal-regulated kinase (MEK)/ERK signaling pathway (76). In addition, miR-23a showed high expression in lung cancer stem cells, and down-regulation of miR-23a could enhance the antitumor effects of erlotinib by increasing PTEN expression to regulate the PI3K/Akt pathway (39).

In addition to the susceptibility of lung cancer patients to MDR during treatment, intrinsic or acquired radioresistance during radiotherapy can limit the overall prognosis of patients. Interestingly, microRNAs associated with the PI3K/AKT signaling pathway have also been shown to play a very important role in radioresistance in lung cancer. For example, some researchers detected different miRNA expression profiles in radiotherapy-sensitive and radiotherapy-resistant patients after surgery for non-small cell lung cancer by microarray, and found that there were 12 abnormally expressed miRNAs in specimens from both groups of patients, and compared with radiotherapy-resistant patients, there were five miRNAs with increased expression in the radiotherapy-sensitive group, with the most obvious upregulation in the expression of miRNA-126, which was confirmed by the subsequent experiments. The miRNA-126 expression was up-regulated in the radiotherapy-sensitive group, and it was confirmed that miRNA-126 inactivated the PI3K/Akt pathway by targeting p-Akt and thus promoted radiation-induced apoptosis (77). In contrast, increased MiR-410 expression has been demonstrated to enhance radioresistance in NSCLC cells through modulating the PTEN/PI3K/mTOR axis (40). Increasing levels of miR-7 in A549 cells has been shown to reduce EGFR and Akt expression, promoting cell responsiveness to radiation therapy (78).

Multidrug resistance (MDR) is an obstacle to successful cancer treatment. The primary mechanism leading to acquired chemoresistance is overexpression of the adenosine triphosphate-binding cassette (ABC) transporter, and numerous studies have confirmed that dysregulation of miRNAs is a critically important cause of this phenotype. Overexpression of the ABC transporters, ABCB1 and ABCG2, are two of the major mediators of MDR in cancer, and antagonists targeting ABCB1 and ABCG2 have undergone clinical Antagonists targeting ABCB1 and ABCG2 have been clinically evaluated; unfortunately, none have been clinically approved. Encouragingly, however, a protein kinase AKT1/2/3 inhibitor, MK-2206, has been shown to reverse the ABCG2-mediated MDR response of lung cancer cells to mitoxantrone, SN-38 and topotecan (116). These findings suggest that in the future, it is possible to block the miRNA-mediated drug-resistant signaling pathway by increasing or decreasing the expression of relevant miRNAs, thus improving the sensitivity of LC cells to therapeutic drugs, providing a new strategy for the treatment of LC patients, and bringing a new hope to LC patients who are in trouble due to drug resistance (**Table 4**).

**Table 4:** Abnormal expression of miRNA-mediated MDR related to PI3K/AKT pathway in lung cancer.

MiRNA	Expression	Target	Resistant drug	Ref.
miR-139-5p	↓	P-AKT	cisplatin	(65)
miR-29c	↓	AKT2	cisplatin	(71)
miR-20a	↓	PI3K	cisplatin	(69)
miR-25-3p	↑	PTEN	cisplatin	(42)
miR-199a-5p	↑	P62	paclitaxel	(36)
miR-107	↓	P-AKT	Paclitaxel	(72)
miR-126	↓	PI3KR2	Adriamycin,vincristine	(47)
miR-17-5p	↓	RRM2	gemcitabin	(45)
miR-34a	↓	PTEN	gefitinib	(49)
miR-522-3p	↑	P-AKT	gefitinib	(37)
miR-30a-5p	↓	P-AKT	gefitinib	(73)
miR-200c	↓	P-AKT	gefitinib	(74)
miR-223	↓	P-AKT	erlotinib	(75)
miR-135a	↑	RAC1	gefitinib	(38)
let-7c	↓	RAS	gefitinib	(76)
miR-23a	↑	PTEN	erlotinib	(39)

↑, Up-regulated expression in lung cancer; ↓,Down-regulated in lung cancer.

### 2.7 Application in the treatment of lung cancer

Early treatment of LC is still primarily surgical, but by the time most patients are diagnosed, they have already missed their chance for surgery. For advanced patients who cannot have their lung cancer surgically removed, the current standard of care consists of 6 weeks of thoracic spine radiotherapy along with dual chemotherapy using either cisplatin or carboplatin. However, with the emergence of drug resistance to these drugs as well as intrinsic or acquired radioresistance, these treatments are becoming less effective (117) (118). Additionally, as molecular biology has developed, a growing number of innovative drugs have been developed that precisely target molecular abnormalities, allowing patients with tumors that have specific genomic aberrations to benefit from molecularly focused therapy. For example, EGFR-mutated NSCLC frequently responds better to treatment with EGFR-TKIs, such as gefitinib and erlotinib, which have significantly improved the outlook for lung cancer patients and given them new hope, but their efficacy has been hampered by the emergence of acquired drug resistance (109). Antibody-targeted therapies against immune checkpoints have shown significant benefits in the treatment of many advanced malignancies, including lung cancer (119).The importance of microRNAs as critical regulatory molecules in radiation, chemotherapy, molecularly targeted therapy, and immunotherapy for lung cancer, among other treatments, has been increasingly demonstrated in recent years. Future molecular biology-based combination therapies for many lung cancer patients (using targeted therapies or immunotherapy) may be the main therapeutic option due to the ongoing discovery of novel microRNA molecular targets, which encourages the development of new therapeutic approaches. Trials in oncology have examined medicines that target the PI3K/AKT pathway (120).

For instance, some research found that miR-142-5p regulates the expression of the proteins PD-L1 and PTEN in CD4+ T cells in NSCLC, which raises the possibility that miR-142-5p could be a potential target for NSCLC treatment (30). MiR-30-5p family interaction with PIK3R2-SIAH2 is considered as a potential therapeutic target for NSCLC, especially LUAD, and SIAH2 is an E3 ubiquitin-protein ligase that mediates the degradation of target proteins (79). MiR-486-5p is downregulated as a tumor suppressor gene in tumor tissues of lung cancer patients, and overexpression of miR-486-5p disrupts the PI3K/Akt pathway and induces CD133+lung tumor stem cells (CSCs) apoptosis, worthy of happiness is that investigators further demonstrated that treatment with cationic lipid nanoparticles encapsulating miR-486-5p mimic (CCL-486) reduced the percentage of CD133+ and inhibited tumor growth in a xenograft tumor model, which offers a novel technique for novel combination therapy (80). Exocrine miR-30b-5p from bone marrow mesenchymal stem cells plays a tumor inhibitory role in NSCLC by inhibiting EZH2 and PI3K/AKT pathways. The results suggest that exocrine from bone marrow mesenchymal stem cells may be used as a new therapeutic strategy for NSCLC (81). Similarly, miR-514b-5p promotes the progress of NSCLC by targeting SGTB through PI 3-K/AKT and p38 signal pathways. The results suggest that miR-514b-5p is expected to become a new target for the diagnosis and treatment of NSCLC (43). It is worth noting that a new study shows that miR-30c enhances the cytotoxicity of NK cells to lung cancer cells by reducing GALNT7 and inactivating PI 3K/AKT pathways. The results of this study reveal that miR-30c may be an effective way to enhance anti-tumor therapy based on NK cells and open up new ideas for the treatment of lung cancer (82).

MiRNAs have also shown good potential in TCM-assisted treatment of lung cancer. For example, baicalein inhibited cell growth by targeting the PTEN/PI3K/Akt pathway via miR-424-3p and increased the sensitivity of NSCLC cells to cisplatin (41). Early studies have shown that curcumin has a variety of effects such as hypotensive, anti-inflammatory, anticholinergic, antioxidant, and antitumor effects. In studies related to lung cancer, curcumin has been shown to exert antitumor effects by inactivating the PI3K/Akt signaling pathway through miR-192-5p (83). Interestingly, another study found that lidocaine inhibits lung cancer cell growth and metastasis via controlling miR-539, which prevents the signaling pathways ERK and PI3K/AKT from being activated (84). Anwuligan (ANW) isolated from nutmeg, also known as myristyl lignan, has been found to have therapeutic potential for a variety of diseases, in lung cancer, the latest studies have confirmed that ANW inhibits the growth and metastasis of NSCLC cells by up-regulating the expression of let-7c-3p (85). Previous studies have confirmed that the reduction of quinone to hydroquinone plays an important role in anti-tumor activity. However, NQO-1 can prevent the reduction of quinones, lead to the accumulation of free radicals and promote tumor progression. Recent lung cancer studies have shown that miR-485-5p can block PI3K/Akt signal pathway and inhibit the growth of LUAD cells by targeting NQO-1 (86).

MiRNAs are not only a very promising predictor of therapeutic sensitivity, but also participate in the whole process of lung cancer therapeutic drug action as key regulators. In-depth study of the mechanism of these endogenous miRNAs in cancer therapy through the PI3K/AKT signaling pathway will help to further elucidate the complex regulatory process *in vivo* and provide a new theoretical basis for the subsequent targeted therapy of related diseases.

## 3 Conclusion

**Figure 3:**
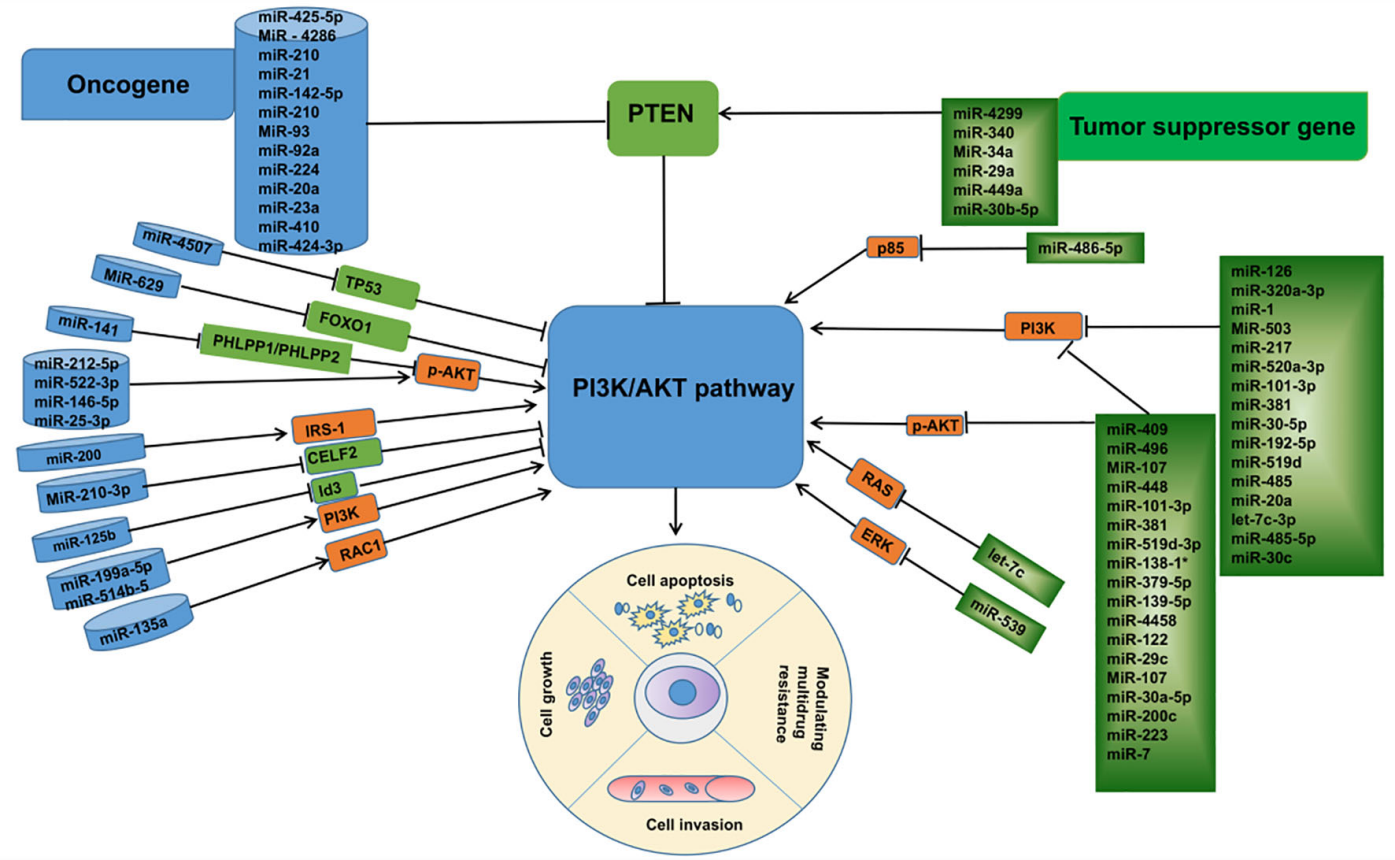
Linkage between microRNAs associated with the PI3K /AKT pathway and their targets in lung cancer.

In summary, combined with a large number of studies in recent years, it is not difficult to find that miRNAs are key regulatory molecules in the process of lung cancer development. These abnormally expressed miRNAs, as oncogenes or tumor genes, affect the biological functions of tumor cells, such as proliferation, migration, apoptosis, invasion, etc., by regulating the expression of proteins related to the PI3K/AKT pathway. (**Figure 3**) In addition, in the treatment process of lung cancer, miRNAs, as important molecules, can not only predict the sensitivity of tumor cells to anticancer drugs, but also regulate the drug resistance phenomenon of lung cancer cells by inhibiting or promoting the expression of their downstream related proteins, among which the miRNA-mediated dysregulation of ABC transporter overexpression should not be ignored, and the discovery of its potential mechanism may provide a theoretical basis for the clinical MDR of cancer The discovery of its potential mechanism may provide a theoretical basis for the clinical treatment of cancer MDR, and the emergence of new gene therapy methods targeting miRNAs to inhibit the ABC transporter has made the reversal of cancer MDR possible, although its clinical application needs to be further investigated. However, with the deepening of related research, local sustained administration of miRNAs based on various novel cationic nanocarriers combined with non-traditional chemotherapeutic drugs will be promising in the future for the inhibition of lung cancer metastasis and the treatment of lung cancer. This brings new hope to lung cancer patients with poor prognosis due to drug resistance, and also provides more theoretical basis for the research and development of molecularly targeted drugs. Although there are still many difficult problems, including many miRNAs have been found to be abnormally expressed in lung cancer, but their functional characteristics and significance still need to be further confirmed; the specific roles of these miRNAs in different subtypes of lung cancer may be different; the miRNAs found to regulate the ABC transporter in chemotherapy-resistant cancers are just the tip of the iceberg of the gene transcripts, and how to combine the miRNAs with anti-cancer drugs for efficient treatment deserves attention. In view of the functional role of miRNAs and their own characteristics, with the deepening of related research, miRNAs are very promising to become a new class of biomarkers, which can play an important value in the early diagnosis, individualized treatment, drug response prediction, and related therapies of lung cancer patients.

## Author contributions

YW: visualization, writing – original draft, writing – review & editing. XH: supervision, writing – review & editing. TZ: writing – review & editing.
